# Can transbronchial lung cryobiopsy benefit adaptive treatment strategies in connective tissue disease-associated interstitial lung disease?

**DOI:** 10.1186/s12890-023-02429-0

**Published:** 2023-04-18

**Authors:** Hideaki Yamakawa, Tamiko Takemura, Shintaro Sato, Makiko Takatsuka, Hiroki Ohta, Tomotaka Nishizawa, Tomohiro Oba, Rie Kawabe, Keiichi Akasaka, Masanobu Horikoshi, Masako Amano, Kazuyoshi Kuwano, Hidekazu Matsushima

**Affiliations:** 1 Department of Respiratory Medicine, Saitama Red Cross Hospital, 1-5 Shintoshin, Chuo-ku, Saitama, 330-8553 Japan; 2 Department of Pathology, Kanagawa Cardiovascular and Respiratory Center, Yokohama, Japan; 3 Department of Respiratory Medicine, Tokyo Jikei University Hospital, Tokyo, Japan; 4 Department of Rheumatology, Saitama Red Cross Hospital, Saitama, Japan

**Keywords:** Connective tissue disease, Interstitial lung disease, Therapy, Transbronchial lung cryobiopsy

## Abstract

**Background:**

Some patients with connective tissue disease (CTD)-associated interstitial lung disease (ILD) progress to pulmonary fibrosis over their disease course despite initial improvement, potentially indicating a poor prognosis. Transbronchial lung cryobiopsy (TBLC) is a new bioptic approach used in diffuse parenchymal lung diseases. This study of CTD-ILD assessed the utility of TBLC in determining therapeutic decision-making strategies.

**Methods:**

We analyzed medical records of 31 consecutive CTD-ILD patients who underwent TBLC focusing on radio-pathological correlation and disease course. A TBLC-based usual interstitial pneumonia (UIP) score was used that assessed three morphologic descriptors: i) patchy fibrosis, ii) fibroblastic foci, and iii) honeycombing.

**Results:**

Among the patients with CTD-ILD, 3 had rheumatoid arthritis, 2 systemic sclerosis, 5 polymyositis/dermatomyositis, 8 anti-synthetase syndrome, 6 Sjögren’s syndrome, and 5 had microscopic polyangiitis. Pulmonary function test results showed a mean %FVC of 82.4% and %DL_CO_ of 67.7%. Among the 10 CTD patients and TBLC-proven pathological UIP, 3 patients had prominent inflammatory cells in addition to a framework of UIP, and pulmonary function of most patients improved with anti-inflammatory agents. Six (40%) of 15 patients with TBLC-based UIP score ≥ 1 had a progressive disease course during follow-up, of whom 4 patients received anti-fibrotic agents.

**Conclusions:**

TBLC in patients with CTD-ILD can help determine an appropriate medication strategy, particularly when UIP-like lesions are present. TBLC may be useful when judging which agents to prioritize, anti-inflammatory or anti-fibrotic, is difficult. Moreover, additional information from TBLC may be beneficial when considering early intervention with anti-fibrotic agents in clinical practice.

## Introduction

Interstitial lung diseases (ILDs) are a heterogenous group of conditions with differing causes. ILD is subdivided into idiopathic interstitial pneumonia, of which idiopathic pulmonary fibrosis (IPF) is one subset; diffuse parenchymal lung diseases, which may be secondary to a variety of occupational or environmental exposures; and multiple rheumatic or connective tissue diseases (CTDs) [1]. The CTDs showing features of ILD include systemic lupus erythematosus, rheumatoid arthritis (RA), systemic sclerosis (SSc), dermatomyositis and polymyositis, Sjögren’s syndrome, and mixed connective tissue disease (MCTD) [1, 2]. CTD-ILD can manifest as a radio-pathological pattern of various ILDs including nonspecific interstitial pneumonia (NSIP), usual interstitial pneumonia (UIP), organizing pneumonia (OP), a fibrosing variant of OP (also known as NSIP + OP), and diffuse alveolar damage [3]. Autoimmune-mediated pulmonary inflammation is considered a key pathobiological pathway in CTD, with anti-inflammatory therapy generally accepted as the cornerstone of treatment for severe and/or progressive CTD-ILD [3, 4]. However, the natural history of CTD-ILD in individual patients can be difficult to predict, and deciding who to treat, when, and with what agent can be challenging [4]. Moreover, regardless of anti-inflammatory therapy, ILD itself can be one of the leading diseases causing a poor prognosis in some patients with CTD [5]. Meanwhile, anti-fibrotic therapy has recently been found to be of benefit in CTD-ILD. The annual rate of decline in forced vital capacity (FVC) was lower in patients with SSc-ILD and progressive fibrosing-ILD (PF-ILD) who received nintedanib as an anti-fibrotic agent compared with placebo in the SENSCIS and INBUILD trials, respectively [6, 7]. Thus, in the therapeutic assessment of CTD-ILD in clinical practice, clinicians are often torn between favoring an anti-inflammatory agent versus an anti-fibrotic agent.

The recently introduced method of transbronchial lung cryobiopsy (TBLC) offers advantages of a higher diagnostic yield than conventional transbronchial lung biopsy, and which have a better safety profile compared with surgical lung biopsy, and thus, the use of TBLC has widened to be included in the diagnostic algorithm of various ILDs [8–10]. Therefore, in the present study, we assessed the utility of TBLC in aiding the therapeutic decision-making strategy in patients with CTD-ILD.

## Materials and methods

We selected 31 consecutive patients with CTD-ILD who underwent TBLC in Saitama Red Cross Hospital between May 2018 and September 2021. These patients with CTD fulfilled each of the standard criteria [11–17]. Six patients who developed manifestations of CTD (i.e., CTD preceded by ILD) during follow-up (median 10 [range 4–30] months) were also included among the 31 patients. This study on humans was conducted according to guidelines of the Declaration of Helsinki and approved by the Ethics Committee of the Medical Research, Saitama Red Cross Hospital, Japan (approval no. 19-C, 23 May 2019). Informed consent was waived by Ethics Committee of the Medical Research, Saitama Red Cross Hospital, Japan because of the retrospective nature of the study.

Baseline clinical measures were obtained within 3 months before the TBLC. Each subject’s radiological findings were reviewed by two expert pulmonologists (S. Sato and H. Matsushima). High-resolution computed tomography (HRCT) patterns were classified as definite UIP, probable UIP, indeterminate for UIP as mixed NSIP/UIP, NSIP, NSIP + OP, or unclassifiable according to our recently modified guidelines for IPF [18, 19]. For example, even if ground-glass opacity was inconspicuous, both central distribution of reticulation with traction bronchiectasis and subpleural reticulations with or without honeycombing in the lower lung were comparable with mixed NSIP/UIP, not NSIP or UIP. For pulmonary emphysema, positive findings of emphysema were visually defined as the presence of an area of low attenuation indicating the lack of a distinct alveolar wall threshold over 10% [20]. In addition, an expert pathologist (T. Takemura) evaluated TBLC specimens for pathological quality, quantity, and confidence according to previous reports [21, 22]. The quality and quantity of the tissue specimens were classified into three grades. Grade A specimens were defined as having an adequate amount of lung tissues corresponding to lesions on HRCT imaging. Grade C specimens were defined as those difficult to evaluate because of small tissue quantity or lack of lesions. Grade B specimens were defined as being between grades A and C. The pathological confidence was also classified into three levels. Definite pathological diagnoses could be made with level A specimens. Diagnoses were difficult with level C specimens. Level B specimens fell between levels A and C, and probable diagnoses could be determined according to a previous study [22]. All adverse events were defined as follows: “serious” adverse events were those with life-threatening consequences; “severe” adverse events were those requiring surgical interventions; and “moderate” and “mild” adverse events were those of bronchial bleeding, with moderate indicated by cold saline or epinephrine being needed three times or more, and mild comprising events other than those indicated above, and pneumothorax, with moderate indicated by the requirement for chest tube placement, and mild comprising events other than those indicated above [22].

Major histologic patterns were classified according to the classification of idiopathic interstitial pneumonias published in 2013 [23]. For the UIP pattern, characteristic histologic features such as lymphoid aggregates with germinal centers or prominent lympho-plasmacytic infiltration indicated “UIP with prominent inflammatory cells” according to previous reports [24, 25]. The mixed NSIP/UIP pattern was defined by topographic similarities with UIP but with no evidence of clear temporal heterogeneity, no or very rare fibroblastic foci, and variable chronic inflammation compared to UIP as noted in a previous report [26]. NSIP + OP was defined as acute inflammatory findings such as OP and alveolar epithelial injury and interstitial cellular infiltration surrounded by hyaline membrane collapse as noted in some previous reports [23, 27, 28]. In addition, for the identification of a UIP pattern, a TBLC-based UIP score was determined according to previously reported criteria [8]. A score of 1, 2, or 3 points was applied based on the presence of one, two, or all three of the following morphologic descriptors: i) patchy fibrosis, ii) fibroblastic foci, and iii) honeycombing [8].

## Results

### Patient characteristics

In our cohort of patients with CTD-ILD (n = 31), 21 patients (67.7%) were female, and 23 patients (74.2%) had a no history of smoking (Table 1). The CTD-ILDs included RA in 3 patients (9.7%), SSc in 3 patients (9.7%), polymyositis/dermatomyositis in 5 patients (16.1%), MCTD in 1 patient (0.3%), anti-synthetase syndrome (ASS) in 8 patients (25.8%), Sjögren’s syndrome in 6 patients (19.3%), and microscopic polyangiitis (MPA) in 5 patients (16.1%). Overall pulmonary function test results showed a mean %FVC of 82.4% and %DL_CO_ of 67.7%.

**Table 1:** Patient characteristics (n = 31)

Characteristic	No. of patients
RA, n (%)	3 (9.7)
SSc, n (%)	3 (9.7)
PM/DM, n (%)	5 (16.1)
MCTD, n (%)	1 (0.3)
ASS, n (%)	8 (25.8)
Sjögren’s syndrome, n (%)	6 (19.3)
MPA, n (%)	5 (16.1)
Female, n (%)	21 (67.7)
Age (years), mean ± SD	66.5 ± 10.3
Never smoker, n (%)	23 (74.2)
Body mass index (kg/m^2^), mean ± SD	23.3 ± 3.7
Emphysema on HRCT, n (%)	2 (6.4)
KL-6 (U/mL), mean ± SD	1354.7 ± 842.2
SP-D (ng/mL), mean ± SD	275.6 ± 234.7
%FVC, mean ± SD	82.4 ± 17.7
%DL_CO_, mean ± SD	67.7 ± 17.9
BAL	
Lymphocytes (%), mean ± SD	22.0 ± 19.7
Neutrophils (%), mean ± SD	10.7 ± 11.3
Eosinophils (%), mean ± SD	5.5 ± 6.3

RA = rheumatoid arthritis; SSc = systemic sclerosis; PM/DM = polymyositis/dermatomyositis; MCTD = mixed connective tissue disease; ASS = anti-synthetase syndrome; MPA = microscopic polyangiitis; SD = standard deviation; KL-6 = Krebs von den Lungen-6; SP-D = surfactant protein-D; FVC = forced vital capacity; DL_CO_ = diffusing capacity of the lungs for carbon monoxide; BAL = bronchoalveolar lavage

### Correlation between HRCT and pathological pattern

The HRCT classification included definite UIP in 5 patients (16.1%), probable UIP in 4 patients (12.9%), mixed NSIP/UIP in 8 patients (25.8%), NSIP in 7 patients (22.6%), NSIP + OP in 6 patients (19.4%), and unclassifiable in 1 patient. The distributions between the HRCT and pathological patterns of TBLC are shown in Table 2. Of 9 patients with definite and probable UIP as indicated by their HRCT pattern, 4 patients (44.4%) had UIP, 1 patient had UIP with prominent inflammatory cells, 1 patient (11.1%) had mixed NSIP/UIP, and 3 patients (33.3%) had NSIP as a pathological pattern. Among 8 patients with an HRCT pattern of mixed NSIP/UIP, UIP was present in 2 patients (25%), UIP with prominent inflammatory cells in 2 patients (25%), mixed NSIP/UIP in 1 patient (12.5%), NSIP in 1 patient (12.5%), and NSIP + OP in 2 patients (25%). Concordance between the HRCT and pathological pattern was relatively high for NSIP (71.4%) and NSIP + OP (83.3%), but 1 patient with NSIP by HRCT had pathological UIP. Pulmonary function of most patients with pathological NSIP or NSIP + OP improved or stabilized for anti-inflammatory agents. Notably, 2 of three patients showing pathological UIP with prominent inflammatory cells stabilized following administration of anti-inflammatory agents (Table 3).

**Table 2:** Distribution of cases according to pattern by HRCT and TBLC specimens (n = 31)

	HRCT pattern					
Pathological pattern of TBLC specimen	Definite UIP	Probable UIP	Mixed NSIP/UIP	NSIP	NSIP + OP	Unclassifiable
	(N = 5)	(N = 4)	(N = 8)	(N = 7)	(N = 6)	(N = 1)
UIP, (N = 7)	2	2	2	1		
UIP with prominent inflammatory cells, (N = 3)	1		2			
Mixed NSIP/UIP, (N = 4)	1		1	1		
NSIP, (N = 10)	1	2	1	5	1	1
NSIP + OP, (N = 7)			2		5	

HRCT = high-resolution computed tomography; TBLC = transbronchial lung cryobiopsy; UIP = usual interstitial pneumonia; NSIP = nonspecific interstitial pneumonia; OP = organizing pneumonia

**Table 3:** Characteristics, HRCT pattern, TBLC diagnosis, and disease course of the 15 CTD-ILD patients with TBLC-based UIP score ≥ 1

TBLC-based UIP score	CTD	HRCT pattern	Pathological diagnosis	Treatment		Changes in PFT results during follow-up period	Disease course	
				Before	After TBLC			
**3** (Patchy fibrosis + FF + HC)	RA	Probable UIP	UIP	ABT + SASP + PSL	TAC added on	(FVC − 1%, %DL_CO_ -7%)/3.2 yrs	Stable	
	MPA (Case 1)	Definite UIP	UIP	-	-	Death (DAH/10 months)		
**2** (Patchy fibrosis + FF)	MPA	Mixed NSIP/UIP	UIP	-	PSL + AZP→PFD added on→Switched to nintedanib	(FVC + 2%, %DL_CO_ -22%)/3.5 yrs	Worsened	
	MPA	Definite UIP	UIP	-	PSL + IVCY, AZP	(FVC − 4%, %DL_CO_ +7%)/2.7 yrs	Stable	
	RA	Definite UIP	UIP with prominent inflammatory cells	-	PSL + TAC	(FVC + 7%, %DL_CO_ -5%)/3.1 yrs	Stable	
	SSc (Case 3)	NSIP	UIP	-	IVCY→Nintedanib added on	(FVC − 24%, %DL_CO_ -5%)/2.6 yrs	Worsened	
	Sjögren’s syndrome	Definite UIP	Mixed NSIP/UIP	-	PFD→Switched to nintedanib	(FVC − 5%, %DL_CO_ -7%)/3.7 yrs	Slightly worsened	
	RA (Case 2)	Mixed NSIP/UIP	UIP with prominent inflammatory cells	-	SASP + TAC→PSL added on	(FVC − 8%, %DL_CO_ -12%)/0.9 yrs	Worsened	
	MPA	Mixed NSIP/UIP	UIP	-	PSL + AZP	(FVC + 14%, %DL_CO_ +14%)/0.6 yrs	Stable	
	Sjögren’s syndrome	Mixed NSIP/UIP	UIP with prominent inflammatory cells	-	PSL + Nintedanib	(FVC + 17%, %DL_CO_ +8%)/0.7 yrs	Stable	
**1** (FF)	ASS	Mixed NSIP/UIP	Mixed NSIP/UIP	-	PSL + TAC	(FVC + 27%, %DL_CO_ +20%)/2.4 yrs	Stable	
	ASS	NSIP	NSIP	-	PSL + TAC	(FVC + 16%, %DL_CO_ +15%)/1.3 yrs	Stable	
	MPA	NSIP	Mixed NSIP/UIP	-	PSL + IVCY, MMF→Nintedanib added on	(FVC − 17%, %DL_CO_ -17%)/1.0 yrs	Worsened	
	Sjögren’s syndrome	Probable UIP	NSIP	-	PSL + MMF	(FVC + 3%, %DL_CO_ +6%)/0.9 yrs	Stable	
**1** (Patchy fibrosis)	Sjögren’s syndrome	Probable UIP	UIP	-	PSL	(FVC − 19%, %DL_CO_ -16%)/2 yrs	Worsened	

HRCT = high-resolution computed tomography; TBLC = transbronchial lung cryobiopsy; CTD = connective tissue disease; ILD = interstitial lung disease; UIP = usual interstitial pneumonia; PFT = pulmonary function tests; RA = rheumatoid arthritis; ABT = abatacept; SASP = salazosulfapyridine; PSL = prednisolone; TAC = tacrolimus; FVC = forced vital capacity; DL_CO_ = diffusing capacity for the lung carbon monoxide; FF = fibroblastic foci; HC = honeycombing; MPA = microscopic polyangiitis; SSc = systemic sclerosis; DAH = diffuse alveolar hemorrhage; NSIP = nonspecific interstitial pneumonia; AZP = azathioprine; PFD = pirfenidone; IVCY = intravenous cyclophosphamide pulse therapy; ASS = anti-synthetase syndrome; MMF = mycophenolate mofetil

**Fig. 1:**
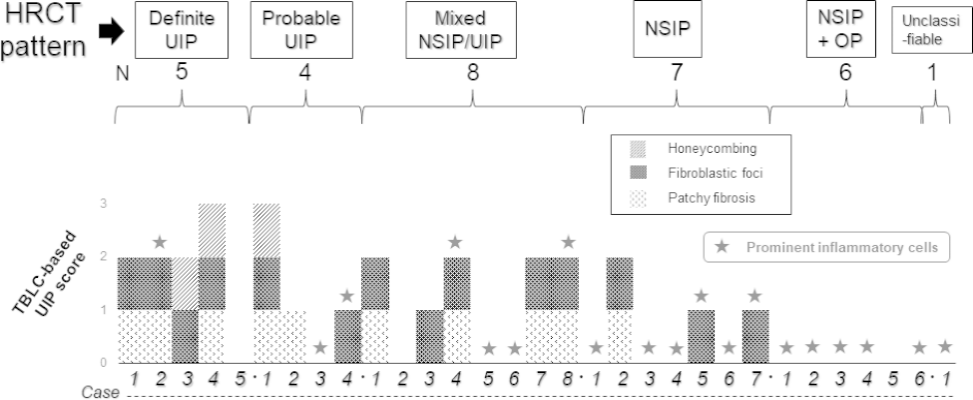
Schematic showing the TBLC-based UIP score according to the three morphologic descriptors of i) patchy fibrosis, ii) fibroblastic foci, and iii) honeycombing and the presence of prominent inflammatory cells in all cases of CTD-ILD

TBLC-based UIP scoring resulted in one patient having a full score (3 points), 3 (60%) of 5 patients with HRCT-definite UIP had a score of 2 points, and 1 patient scored zero points (Fig. 1). Among the 4 patients with probable UIP on HRCT, one patient had a full score (3 points), 2 patients scored 1 point, and one patient scored zero points. TBLC specimens of 3 (33.3%) of 9 patients with definite or probable UIP on HRCT showed prominent inflammatory cells. In the 8 patients with mixed NSIP/UIP on HRCT, 4 patients (50%) had scores of 2, and 1 patient had a score of 1. Among the 7 patients with HRCT-identified NSIP, one patient (14.2%) had a score of 2, and 2 patients (28.6%) had a score of 1. None of the 6 patients with NSIP + OP on HRCT had a TBLC-based UIP score.

### Treatment response and outcome during follow-up in patients with TBLC-based UIP score ≥ 1

**Fig. 2:**
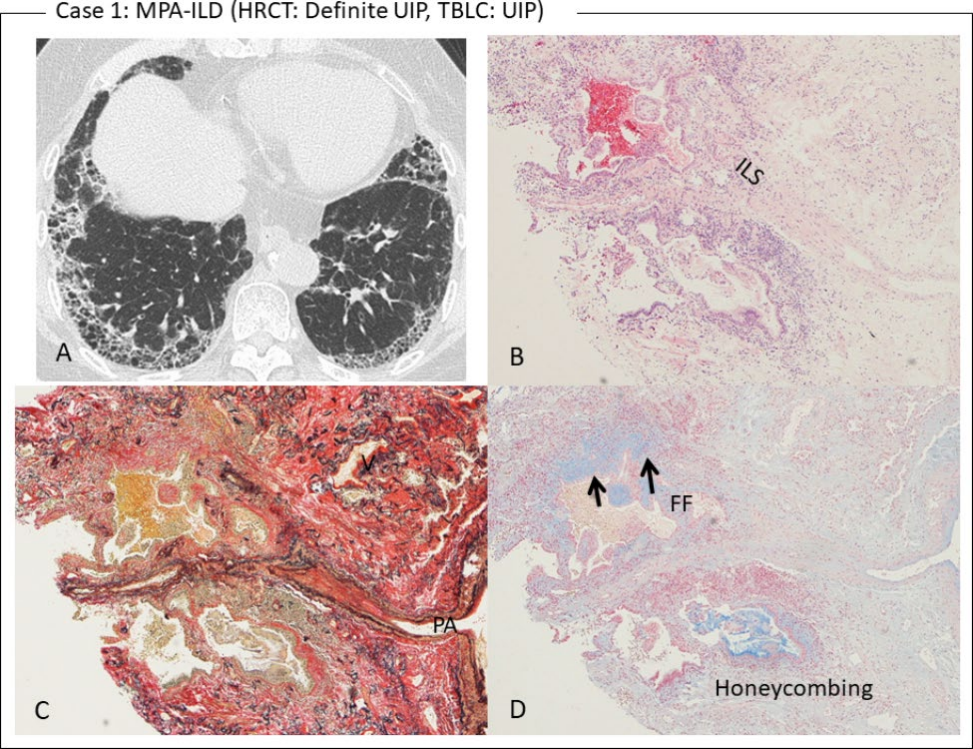
Case 1. **(A)** HRCT showed a definite UIP pattern in a patient with MPA. **(B)** Cystic peripheral airspaces apposed to the interlobular septum (ILS) are shown (hematoxylin-eosin stain, ×4). **(C)** A UIP pattern appearing as peri-lobular/patchy fibrosis with cystic airspaces similar to honeycombing is shown (Elastica van Gieson stain, ×4). **(D)** Alcian blue stain (×4) revealed fibroblastic foci (FF, arrows) in the cystic airspaces

Characteristics, HRCT pattern, TBLC diagnosis, and disease course of the 15 patients with TBLC-based UIP score ≥ 1 are summarized in Table 3. Among the 2 patients with a TBLC-based UIP score of 3, one patient (RA-ILD) had been stable for 3.2 years, but the other patient (case 1) died 10 months after the initial diagnosis of ILD from severe complications of diffuse alveolar hemorrhage as a development of MPA (i.e., CTD preceded by ILD) without medical intervention. The same patient (Fig. 2) with MPA-ILD showed a HRCT-definite UIP pattern at the initial diagnosis of ILD. TBLC showed structural modification abruptly alternating with relatively normal areas of the lung as patchy fibrosis, fibroblastic foci, and honeycombing. There were no findings of vasculitis.

**Fig. 3:**
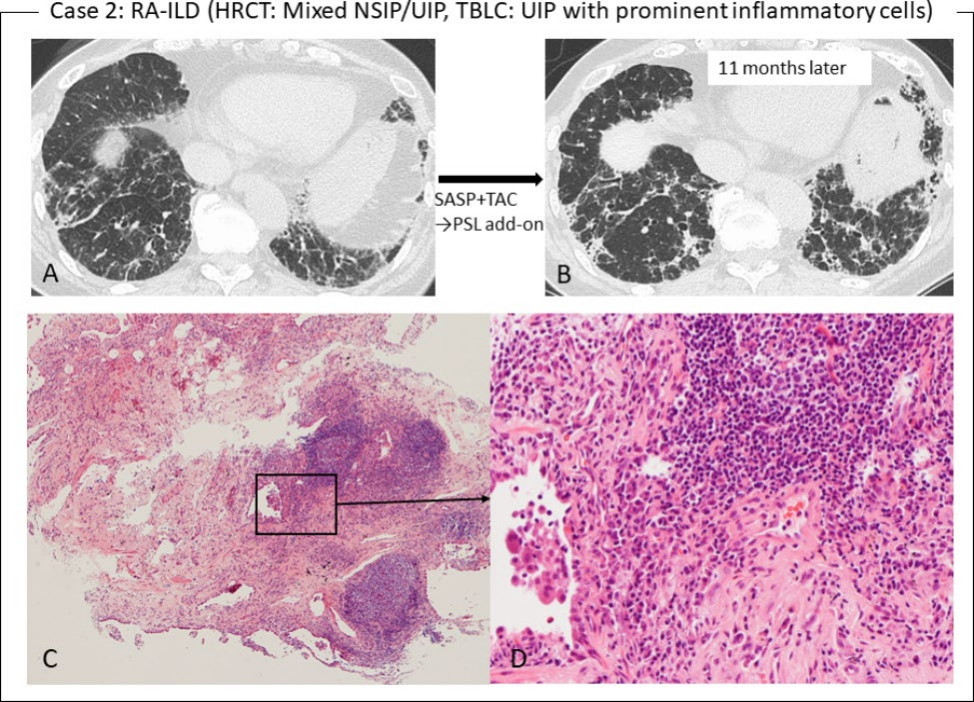
Case 2. **(A)** HRCT showed a mixed NSIP/UIP pattern with RA-ILD, which have both central distribution of reticulation or ground-glass opacity as the component of NSIP and subpleural reticulations as the component of UIP. **(B)** After 11 months of treatment with anti-inflammatory agents salazosulfapyridine (SASP) and tacrolimus (TAC), the extent of disease had increased. **(C)** Pathological findings of TBLC showed that peri-lobular fibrosis and lymphoid follicles with a germinal center are present in the fibrous area close to the interlobular septum (hematoxylin-eosin stain, ×4). **(D)** Plasma cells and infiltration by lymphocytes are shown in a magnified square from panel C (hematoxylin-eosin stain, ×20)

**Fig. 4:**
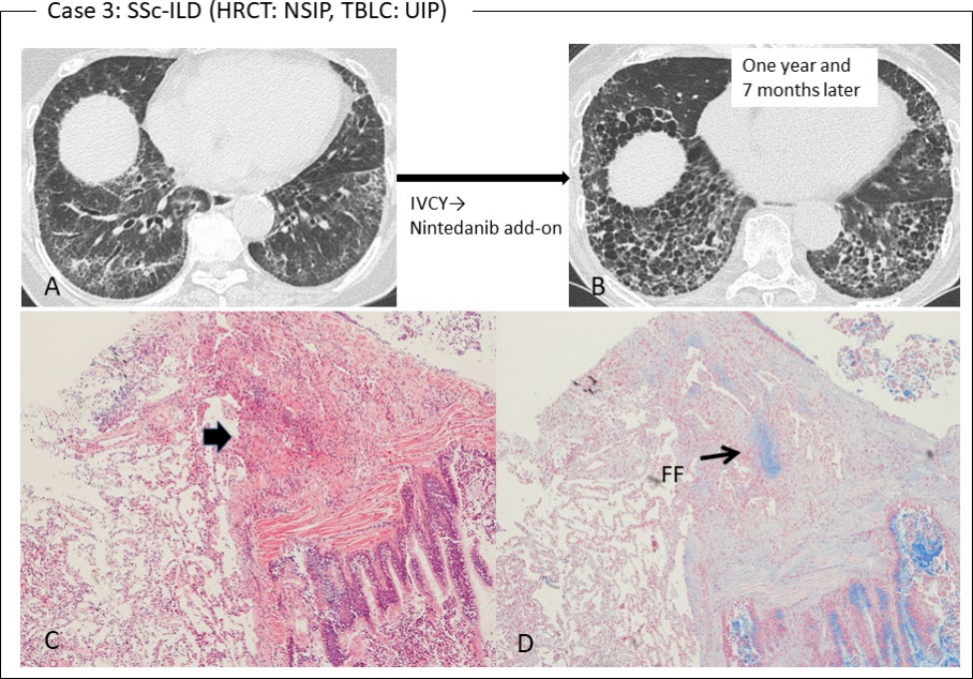
Case 3. **(A)** HRCT showed a NSIP pattern with SSc-ILD. **(B)** At 1 year and 7 months of cyclophosphamide (IVCY) therapy, the disease area apparently increased, manifesting as progressive fibrosis, and the patient started on treatment with an additional agent, nintedanib. **(C)** Pathological finding of TBLC showed patchy fibrosis adjacent to the bronchial wall (arrow) on a background of normal alveoli (hematoxylin-eosin stain, ×4). **(D)** Alcian blue stain (×4) focused on the fibrosis revealed small fibroblastic foci (FF, arrow)

Among the 8 patients with a TBLC-based UIP score of 2, 4 patients had a stable disease course during follow-up, whereas the other 4 patients worsened or showed slight deterioration (N = 1). For the latter, for example, the patient (case 2) with RA-ILD had a HRCT pattern of mixed NSIP/UIP and pathological UIP with prominent inflammatory cells (Fig. 3). TBLC showed UIP with prominent inflammatory cells, which manifested as plasmacyte infiltration and the presence of lymphoid follicles with a germinal center in the fibrous area close to the interlobular septum. After 11 months of anti-inflammatory therapy with salazosulfapyridine and tacrolimus, HRCT showed an increase in disease extent, and prednisolone was added to the patient’s therapy. The other patient (case 3) with SSc-ILD had a HRCT pattern of NSIP (Fig. 4). However, the TBLC specimen showed patchy fibrosis and fibroblastic foci as the pathology of the UIP. The patient suffered a progressive course despite having received intravenous cyclophosphamide pulse therapy followed by nintedanib as an anti-fibrotic agent. In addition, the other patient with MPA-ILD also had a progressive course despite anti-inflammatory therapy (prednisolone/azathioprine) and anti-fibrotic therapy (pirfenidone/nintedanib).

Five patients had a TBLC-based UIP score of 1. Two patients worsened during follow-up, with one of them with MPA-ILD receiving nintedanib added on to anti-inflammatory agents such as prednisolone, cyclophosphamide pulse therapy, and mycophenolate mofetil. The other 3 patients had a stable disease course.

### Treatment response and outcome during follow-up in patients with TBLC-based UIP score = 0

Among the 16 patients with a TBLC-based UIP score of 0, the CTD-ILDs included ASS in 6 patients, PM/DM in 5 patients, SSc in 2 patients, Sjögren’s syndrome in 2 patients, and MCTD in one patient. As pathological patterns of TBLC, 7 patients had NSIP + OP and 9 patients had NSIP. Of these 16 patients, 13 could be evaluated for changes in pulmonary function, and all of these patients received anti-inflammatory agents such as prednisolone, tacrolimus, and mycophenolate mofetil after TBLC. During the follow-up period (median 1.5 [range 0.5–3.5] years) of these 13 patients, 11 (84.6%) had a stable course, whereas the other 2 patients (15.4%) had a progressive course, with one of them with ASS-ILD receiving nintedanib added on to anti-inflammatory agent therapy. Of the 3 patients whose disease course could not be evaluated for changes in pulmonary function, one patient had a stable course as shown by HRCT and pulmonary symptoms controlled with anti-inflammatory agents. The other 2 patients died within 6 months after TBLC due to causes unrelated to ILD.

### Pathological evaluation and adverse events by TBLC

In most patients, 2 (N = 14, 45.2%) or 3 (N = 16, 51.6%) TBLC pathological samples could be obtained (Table 4). The percentages of specimens classified as grades A and B, which were determined to have value for evaluation, were 83.9% and 16.1%, respectively. Definite and probable pathological diagnoses (levels A and B) were made in 67.7% and 29.0%, respectively. Level C indicating poor confidence was assessed in 3.2% of the patients. No serious complications were identified among the adverse events. Bronchial bleeding was identified as mild in 19.4% and as moderate in 12.9% of patients. Mild pneumothorax was identified in 1 patient (3.2%). No patients required drug therapy or needed oxygen therapy for > 24 h as a result of TBLC-related pneumonia or respiratory failure.

**Table 4:** Pathological evaluation and adverse events by TBLC

Total	No. of patients, n = 31
	n (%)
Number of samples	
1	1 (3.2)
2	14 (45.2)
3	16 (51.6)
Pathological quality and quantity	
Quality score	
Grade A	26 (83.9)
Grade B	5 (16.1)
Grade C	0 (0.0)
Pathological confidence	
Level A	21 (67.7)
Level B	9 (29.0)
Level C	1 (3.2)
Adverse events	
Bronchial bleeding	
Mild	6 (19.4)
Moderate	4 (12.9)
Pneumothorax	
Mild	1 (3.2)
Moderate	0 (0.0)

TBLC = transbronchial lung cryobiopsy

The quality and quantity (i.e., less than or equal to both grade B and level B) of the TBLC samples of 5 patients could not be sufficiently proven. Four patients had a TBLC-based UIP score ≥ 1; 2 patients had a progressive course; one patient with RA-ILD (TBLC-based UIP score of 3, grade B, level B) had a stable course for 3.2 years (Table 3); and one patient with MPA-ILD (TBLC-based UIP score = 2, grade B, level B) also had stable course for 0.7 years. In contrast, one patient with ASS-ILD and a TBLC-based UIP score of 0 (grade B, level C) had a progressive course, and this same patient received nintedanib added on to anti-inflammatory agents.

## Discussion

Until now, only one other study, which included 14 patients with CTD-ILD, has reported the utility of TBLC for CTD-specific ILD [29]. That study concluded that TBLC can add extra diagnostic value by effectively identifying specific types of histology for patients with CTD-ILD [29]. In contrast, we investigated 31 cases of CTD-ILD histologically confirmed by TBLC and also determined whether adding histological information is useful in developing a therapeutic decision-making strategy. The findings of the present study have important implications.

First, in the case of TBLC-proven pathological UIP in patients with CTD, some patients also have prominent inflammatory cells in addition to a framework of UIP. Pulmonary function of these patients improved with anti-inflammatory agents because lymphocyte aggregation in the interstitium and lymphoid follicles with germinal centers are more frequently observed in CTD-UIP than in IPF [5]. Moreover, in some CTD-ILD cases, it is difficult to classify imaging findings by HRCT as UIP or NSIP, and, in fact, a certain number of patients in the present study were classified as having mixed NSIP/UIP. These features (i.e., UIP with prominent inflammatory cells, mixed NSIP/UIP) may be associated with a better response to anti-inflammatory agents. Therefore, TBLC may be useful when it is difficult to judge which should be prioritized, anti-inflammatory or anti-fibrotic agents.

Second, pathological evaluation using a TBLC-based UIP score may be useful as a predictor of PF-ILD. Cases of UIP showing a combination of patchy fibrosis and fibroblastic foci, with or without honeycombing, were assigned a rating of high confidence in the UIP diagnosis [8]. A pattern of UIP develops in a substantial subset of patients with other non-idiopathic pulmonary fibrotic ILDs and may be associated with higher risk of PF-ILD compared with other fibrotic morphological patterns. Knowledge of an underlying baseline UIP pattern can help to identify a higher likelihood of progression in patients with worsening symptoms or radiological findings [30] because the later phase of fibrogenesis, which is thought to be shared by all conditions with any etiology such as CTD, leads to lung tissue remodeling and subpleural fibrotic lesions as UIP [31]. The INBUILD study showed the effectiveness of nintedanib as an anti-fibrotic agent on PF-ILD other than IPF. That study included a range of fibrosing ILDs, incorporating 170 patients with CTD-ILD, including RA-ILD, SSc-ILD, and MCTD-ILD [8]. In the present study, 6 (40%) of the 15 patients with a TBLC-based UIP score ≥ 1 showed a progressive disease course during the follow-up period, and 4 of them received anti-fibrotic agents (Table 3). If a patient has a TBLC-based UIP score ≥ 1, we speculate that this finding (such as having a UIP-like lesion or not) can lead to considering early intervention with an anti-fibrotic agent as long-term treatment. As mentioned in the previous paragraph, although patients with inflammatory cells in a framework of UIP may potentially respond to anti-inflammatory agents in the short term, they face the possibility of a progressive disease course in the long run. Taken together, although we definitively addressed this, additional information from TBLC may be beneficial when considering early intervention with anti-fibrotic agents in patients with a UIP-based score.

Third, most (5/6) of the patients with NSIP + OP on HRCT were also consistently proven pathologically to have NSIP + OP. Because anti-inflammatory agents are the primary treatment for NSIP + OP [5], when NSIP + OP is present on HRCT, low priority should be placed on performing TBLC. However, because of frequent recurrence and/or refractoriness to anti-inflammatory agents, some patients progress to pulmonary fibrosis over their disease course despite initial improvement [5]. Therefore, if the process is still ongoing, TBLC may be useful only when it is difficult to judge which is the dominant condition during the middle of anti-inflammatory treatment: inflammation or fibrosis.

As adverse events, bleeding and pneumothorax are the most common complications of TBLC [9, 10, 22]. In our study, no patients with severe or serious bleeding or serious pneumothorax were identified. However, previously published data on moderate or severe bleeding varied widely from 1.7 to 15.7%, and that on pneumothorax requiring drainage ranged from 0.9 to 15.5% [22]. As reported by Wu et al. [29], we also placed an endobronchial balloon for bronchial blockage as a routine procedure in our patients, and our cohort included only those with mild (not severe) disease, which might have led to a low rate of, and milder, complications. Importantly, attention must be paid to the quality and quantity of the pathological specimen, which could not be sufficiently proven in TBLC samples of 5 patients in the present study. In fact, one patient with a UIP score of 3 had a long-term stable course, whereas another with a UIP score of 0 had a progressive course. In other words, these patients had clinical courses contradictory to those indicated by their TBLC-UIP scores. When TBLC specimens are inadequate, clinicians should not place too much emphasis on pathological findings in the therapeutic assessment of CTD-ILD.

Our study has several limitations. First, it is a single-institution, retrospective study with a small sample size, and there was variation of follow-up period with each patient. Second, there is selection bias because only those patients who could undergo TBLC were included as a pulmonologist determined the necessity for the procedure and enrollment into the study. Third, because the protocol for ILD diagnosis is not standardized in our hospital, clinical practice varies between different clinicians, such as pulmonologists and rheumatologists. Therefore, these data may mask potential differences in care. Fourth, expert thoracic radiologists should analyze HRCT findings, not pulmonologists. Although it is common for pulmonologists to perform HRCT analysis in clinical practice, whether analysis by thoracic radiologists is preferable will require further study. Fifth, a comparable analysis could not be performed in the present study. However, we believe that judgement of whether a patient has UIP-like lesions is meaningful when determining a strategy for treatment because clinicians should consider the possibility that patients will develop progressive ILD in the long run if they have UIP-like lesions at the time of diagnosis [5, 8, 25]. Although the present study is descriptive and no statistical analysis was performed, we believe that it provides useful information on TBLC in patients with CTD-ILD.

## Conclusions

Based on our experience, TBLC in patients with CTD-ILD can be helpful in determining an appropriate medication strategy, particularly in those with a UIP-like lesion, because a framework of UIP is frequently observed in PF-ILD of any etiology, which can indicate a poor prognosis. When deciding which anti-fibrotic agents are approved for PF-ILD including CTD-ILD, the additional information provided by TBLC may lead to considering early intervention with anti-fibrotic agents in clinical practice. Additional studies with a larger sample size will be needed to better determine the therapeutic decision-making strategies in CTD-ILD.

## Data Availability

The datasets used and/or analyzed during the current study are available from the corresponding author on reasonable request.

## Abbreviations

ASS: anti-synthetase syndrome
CTD: connective tissue disease
HRCT: high-resolution computed tomography
ILD: interstitial lung disease
IPF: idiopathic pulmonary fibrosis
MCTD: mixed connective tissue disease
MPA: microscopic polyangiitis
NSIP: nonspecific interstitial pneumonia
OP: organizing pneumonia
PF-ILD: progressive fibrosing-ILD
RA: rheumatoid arthritis
SSc: systemic sclerosis
TBLC: transbronchial lung cryobiopsy
UIP: usual interstitial pneumonia
